# Plasma D-dimer concentration in patients with systemic sclerosis

**DOI:** 10.1186/1477-9560-4-2

**Published:** 2006-01-18

**Authors:** Giuseppe Lippi, Alessandro Volpe, Paola Caramaschi, Gian Luca Salvagno, Martina Montagnana, Gian Cesare Guidi

**Affiliations:** 1 Istituto di Chimica e Microscopia Clinica, Dipartimento di Scienze Morfologico-Biomediche, Università degli Studi di Verona, Verona, Italy; 2 Dipartimento di Medicina Clinica e Sperimentale, Università degli Studi di Verona, Verona, Italy

## Abstract

**Background:**

Systemic sclerosis (SSc) is an autoimmune disorder of the connective tissue characterized by widespread vascular lesions and fibrosis. Little is known so far on the activation of the hemostatic and fibrinolytic systems in SSc, and most preliminary evidences are discordant.

**Methods:**

To verify whether SSc patients might display a prothrombotic condition, plasma D-dimer was assessed in 28 consecutive SSc patients and in 33 control subjects, matched for age, sex and environmental habit.

**Results and discussion:**

When compared to healthy controls, geometric mean and 95% confidence interval (IC95%) of plasma D-dimer were significantly increased in SSc patients (362 ng/mL, IC 95%: 361–363 ng/mL vs 229 ng/mL, IC95%: 228–231 ng/mL, p = 0.005). After stratifying SSc patients according to disease subset, no significant differences were observed between those with limited cutaneous pattern and controls, whereas patients with diffuse cutaneous pattern displayed substantially increased values. No correlation was found between plasma D-dimer concentration and age, sex, autoantibody pattern, serum creatinine, erythrosedimentation rate, nailfold videocapillaroscopic pattern and pulmonary involvement.

**Conclusion:**

We demonstrated that SSc patients with diffuse subset are characterized by increased plasma D-dimer values, reflecting a potential activation of both the hemostatic and fibrinolytic cascades, which might finally predispose these patients to thrombotic complications.

## Background

Systemic sclerosis (SSc) is an autoimmune disorder of the connective tissue characterized by widespread vascular lesions and fibrosis. In SSc, vasospasm causes frequent episodes of reperfusion injury and free radical-mediated endothelial dysfunction, which might finally influence the onset of local thrombotic complications. The characteristic vascular involvement affects primarily small arteries and capillaries, causing reduced blood flow and tissue ischemia and supporting the typical clinical manifestations of this unique autoimmune disorder [1]. However, mechanisms involved in the endothelial injury are as yet elusive and most biochemical evidences are often inconclusive or controversial. Some earlier investigations suggested that SSc patients might be characterized by a procoagulant state, reporting depressed basal and stimulated fibrinolytic activity, while others studies have reported normal plasma fibrinolytic activity and normal skin and plasma tissue plasminogen activator (tPA) levels [2-4]. It has been also reported that the lack of a consistent and homogenous increase of some fibrinolytic markers, in the presence of normal levels of antithrombin, might indirectly highlight an impairment of the heparan sulphate-antithrombin system, which would finally promote thrombin generation [3]. Conversely, Cerinic and colleagues provided evidence that fibrinolysis might be impaired in SSc, as shown by reduced D-dimer and decreased levels of plasminogen activator inhibitor [4]. In synthesis, there are no conclusive evidences on the activity of the hemostatic and fibrinolytic pathways in SSc so far.

D-dimer, a breakdown product of cross-linked fibrin, was proven useful for the diagnostic evaluation of several thrombotic disorders. Moreover, an increased D-dimer value in plasma is a reliable marker of a systemic prothrombotic state, likely superior to alternative fibrinolytic markers, and its measurement might be helpful in predicting or preventing thrombotic events in the single patient [5]. Therefore, to investigate whether SSc patients might be characterized by a potential prothrombotic condition, plasma D-dimer vales were measured in a subset of SSc patients, compared with those of a healthy matched control population and further associated with SSc disease subset.

## Methods

Plasma D-dimer was measured in 28 consecutive SSc patients (2 males and 26 females; mean age 50 ± 15 years, 17 with limited and 11 with diffuse disease patterns), who fulfilled the American Rheumatism Association's criteria for the diagnosis of SSc [6] and in 33 control subjects, matched for age (48 ± 13 years), sex (3 males, 30 females) and environmental habit, recruited among healthy hospital personnel. Samples were collected in the morning; all subjects were in a fasted state. The research was carried out according to the principles of the Declaration of Helsinki and an informed consent for testing was received from all individuals recruited to the study. Blood was collected after an overnight fast into siliconized vacuum tubes, containing 0.105 mol/l sodium citrate (Becton-Dickinson, Oxford, UK). Samples were gently mixed and centrifuged for 10 min at 15°C at 1500 × *g*; plasma was separated and stored in aliquots at -70°C until measurement. Plasma D-dimer was measured employing Vidas DD, a rapid and quantitative automated enzyme linked immunosorbent assay with fluorescent detection, on the Mini Vidas immunoanalyzer (bioMerieux, Marcy l'Etoile, France). Analytical imprecision, expressed in terms of mean inter-assay coefficient of variation (CV), was quoted by the manufacturer as being lower than 5%. Significance of differences between samples was assessed, following logarithmic conversion of data, by parametric tests (Student's t-test, ANOVA test, Pearson's correlation); the level of statistical significance was set at p < 0.05.

## Results and discussion

When compared to healthy controls, geometric mean and 95% confidence interval (IC95%) of plasma D-dimer concentration appeared significantly increased in SSc patients (362 ng/mL, IC 95%: 361–363 ng/mL vs 229 ng/mL, IC95%: 228–231 ng/mL, p = 0.005). After stratifying SSc patients according to disease subset, no significant differences were observed between those with limited cutaneous pattern (lcSSc) and controls (geometric mean plasma D-dimer: 283 ng/mL, IC95%: 282–285 ng/mL; p = 0.61), whereas patients with diffuse cutaneous pattern (dcSSc) displayed substantially increased values (geometric mean plasma D-dimer: 538 ng/mL, IC95%: 536–539 ng/mL; p < 0.001). Additionally, patients with active disease, as evaluated according to the European Scleroderma Study Group criteria [7], displayed higher D-dimer levels as compared to patients with inactive disease (p = 0.027). As further shown in table 1, D-dimer concentration correlated significantly with the modified Rodnan total skin score (TSS) and the forced vital capacity (FVC). No correlation was observed between plasma D-dimer concentration and age, sex, autoantibody pattern, serum creatinine, erythrosedimentation rate, nailfold videocapillaroscopic pattern and pulmonary involvement, ascertained according to the score proposed by Medsger et al [8].

**Table 1:** Plasma D-dimer values and association with clinical features in 28 patients with systemic sclerosis (SSc) and 33 healthy matched controls (values are expressed as geometric mean and 95% interval of confidence).

		D-dimer (ng/mL)	p
Patients subset	Controls (n = 33)	229 (228–231)	0.005
	SSc patients (n = 28)	362 (361–363)	
Clinical subset	Limited SSc (n = 17)	283 (282–285)	0.015
	Diffused SSc (n = 11)	538 (536–539)	
Disease activity	Inactive SSc (n = 20)	306 (305–308)	0.027
	Active SSc (n = 8)	562 (560–564)	
Total skin score		r = 0.63*	<0.001
Forced vital capacity		r = 0.45*	0.016

* Pearson's correlation coefficient.

The pathogenesis of the endothelial injury in SSc is as yet elusive and most biochemical evidences are often inconclusive or controversial. Although endothelial cell apoptosis and impaired angiogenesis have received major attention among the mechanisms involved in the characteristic vascular dysfunction, recent studies provided clear evidence of a significant activation of the coagulation cascade, resulting in a procoagulant state that might finally raise the relative risk of thrombotic events in these patients. In SSc, the peculiar vascular lesions and fibrosis were claimed to impair endothelial function, as suggested by impairment of fibrinolysis and activation of the coagulation pathway. The following loss of the balance between fibrinolysis and coagulation might finally contribute to vessel engulfment with fibrin and breakdown of vessel patency, symptomatic of a tendency to the development of thrombotic complications in this particular autoimmune disorder [4]. D-dimer is a heterogeneous class of end-stage degradation products that directly reflect the level of lysed cross-linked fibrin, occurring *in vivo *with a wide range of molecular weights. Therefore, D-dimer is a well-recognized marker of a systemic prothrombotic state [5,9] and appears a strong, consistent predictor of cardiovascular events in the general population, in patients with cardiovascular disease and in other pathologies characterized by an increased risk of thrombosis [10-12]. Accordingly, D-dimer measurement could be reliably used as an initial screening test in patients with clinically suspected thrombosis, as its high negative predictive value enables to validly rule out ongoing thrombotic complications [12]. Little is known on the thrombotic tendency of SSc patients so far [13]. At variance with previous investigations [2-4], we demonstrated that SSc patients with diffuse subset are characterized by increased plasma D-dimer values, reflecting a potential activation of both the coagulation and fibrinolytic pathways.

## Conclusion

Although increased D-dimer values in SSc patients were occasionally observed in earlier studies, the association between plasma D-dimer and disease subset is likely an original and innovative issue. The significant correlation observed with disease activity, cutaneous involvement and forced vital capacity, further suggests that SSc patients, especially those with diffuse subset, display a hypercoagulable state, which might finally predispose this peculiar subset of patients to the development of thrombotic complications.

## Authors' contributions

GL: conceived of the study, participated in its design and coordination and drafted the manuscript; AV: participated in the design of the study, performed the statistical analysis and helped to draft the manuscript; PC: participated in the design and coordination of the study; GLS: participated in the design of the study; MM: participated in the design and coordination of the study and performed the measurement; GCG: participated in the design and coordination of the study. All authors read and approved the final manuscript. The authors declare that they have no competing interests.
